# A Study of Cytological Changes in the Bone Marrow of Patients with Severe Fever with Thrombocytopenia Syndrome

**DOI:** 10.1371/journal.pone.0083020

**Published:** 2013-12-12

**Authors:** Xing QuanTai, Chen FengZhe, Song XiuGuang, Chen DongGe

**Affiliations:** 1 Department of Infectious Diseases , Qilu Hospital of Shandong University, Jinan, China; 2 Infectious Diseases Hospital of Jinan, Jinan, China; 3 Department of Blood Laboratory, Qilu Hospital of Shandong University, Jinan, China; Rutgers - New Jersey Medical School, United States of America

## Abstract

**Background:**

Peripheral blood leucopenia and thrombocytopenia are the main manifestations in severe fever with thrombocytopenia syndrome (SFTS) patients. However, the underlying causes are poorly understood. Therefore, we aimed to investigate cytology of bone marrow samples collected from SFTS patients.

**Methods:**

10 SFTS patients were identified by typical clinical manifestations, detection of peripheral blood leucopenia and thrombocytopenia, and nucleic acid-based detection of the newly identified bunyavirus. SFTS patients, along with 10 participants with acute aplastic anemia and 10 healthy volunteers were enrolled in this study after written informed consent to undergo bone marrow cytological examination.

**Results:**

We observed similar bone marrow properties in SFTS patients and healthy volunteers, significantly different from the characteristics observed in acute aplastic anemia patients.

**Conclusion:**

Similarities between bone marrow samples collected from SFTS patients and healthy volunteers suggest that peripheral blood leucopenia and thrombocytopenia do not result from bone marrow cell plasticity.

## Introduction

 Recently, patients presenting with severe fever, digestive and respiratory symptoms accompanied by laboratory test results indicative of thrombocytopenia and leucopenia, were identified in rural regions of China, with some dying from multiple organ failure. This disease was designated severe fever with thrombocytopenia syndrome (SFTS) based on its clinical characteristics. In most cases, patients’ white blood cell and platelet counts were progressively reduced. However, administration of bone marrow-stimulating medications was seldom efficacious, while supplementation with exogenous platelets only temporarily maintained platelet levels. In an effort to unveil the mechanisms underlying the development of thrombocytopenia and leucopenia in SFTS patients, we compared these patients to acute aplastic anemia patients and healthy individuals for cytological properties of bone marrow samples.

## Subjects and Methods

### Source of samples

Whole blood and bone marrow samples from 10 SFTS patients (6 men and 4 women aged 30–67 [48.7 ± 11.2]) were obtained from the Departments of Infectious Disease and Hematology of Qilu Hospital, Shandong University and Jinan Infectious Diseases Hospital. Baseline characteristics and clinical outcomes are shown in Table 1. Samples from 10 patients with acute aplastic anemia (4 men and 6 women aged 28–54 [42.1 ± 9.6]) were obtained from the Hematology Department, Qilu Hospital. It is well known that aplastic anemia reduces white and red blood cells as well as platelets in peripheral blood, similar to SFTS. The mechanism of blood cell reduction in aplastic anemia is well known: altered production of myeloid cells. Therefore, we chose samples from acute aplastic anemia as positive controls to unveil the mechanisms of SFTS. Baseline characteristics of these subjects are shown in Table 2. Samples from 10 healthy volunteers (4 men and 6 women aged 25–52 [40.9±8.8]) were obtained from Shandong University. Baseline characteristics are shown in Table 3. No significant age differences were found among the 3 groups (P>0.10).

**Table 1:** Baseline characteristics of SFTS patients.

Patient no.	Gender	Age(years)	Total WBCcount(×10^9^/L)	Neu	Lym	Mon	Eos	Bas	RBC count (×10^12^/L)	Platelet count(×10^9^/L)	Clinical outcomes
1	Male	37	1.63	1.12	0.34	0.12	0.05	0.00	4.33	22	Cured
2	Female	44	2.44	1.65	0.48	0.18	0.08	0.05	4.65	35	Cured
3	Male	56	1.92	0.90	0.84	0.13	0.05	0.02	5.06	57	Died
4	Male	49	0.83	0.53	0.20	0.12	0.01	0.00	4.17	18	Died
5	Female	50	3.21	2.45	0.62	0.10	0.02	0.02	3.81	26	Cured
6	Male	30	4.35	2.48	1.42	0.31	0.14	0.00	4.43	36	Cured
7	Male	67	2.49	1.30	1.04	0.10	0.03	0.03	5.17	39	Cured
8	Female	48	1.74	1.27	0.25	0.12	0.06	0.04	3.92	24	Died
9	Male	43	3.08	2.08	0.86	0.08	0.06	0.00	4.34	27	Cured
10	Female	63	2.16	1.50	0.34	0.23	0.07	0.02	4.05	30	Cured
mean±SD		48.7±11.2	2.39±0.98	1.53±0.65	0.64±0.40	0.15±0.07	0.06±0.04	0.02±0.02	4.39±0.45	31.4±11.2	

WBC: white blood cell; Neu: neutrophil; Lym: lymphocyte; Mon: monocyte; Eos: eosinophil; Bas: basophile; RBC: red blood cell

**Table 2:** Baseline characteristics of acute aplastic anemia patients.

Patient no.	Gender	Age(years)	Total WBC count(×10^9^/L)	Neu	Lym	Mon	Eos	Bas	RBC count (×10^12^/L)	Platelet count(×10^9^/L)
1	Male	28	1.82	0.84	0.80	0.11	0.05	0.02	2.41	36
2	Male	38	1.88	1.02	0.51	0.22	0.08	0.05	1.62	25
3	Female	46	2.83	1.39	1.35	0.06	0.03	0.00	3.08	47
4	Female	54	1.27	0.67	0.36	0.13	0.08	0.03	2.78	24
5	Male	36	2.23	1.46	0.57	0.18	0.01	0.01	2.34	36
6	Female	50	3.28	2.06	0.72	0.32	0.10	0.08	2.12	54
7	Male	49	1.52	0.68	0.56	0.18	0.05	0.05	3.17	19
8	Female	38	0.69	0.32	0.28	0.08	0.01	0.00	2.73	10
9	Male	53	2.11	1.26	0.66	0.13	0.04	0.02	1.76	27
10	Male	29	0.63	0.36	0.25	0.02	0.00	0.00	1.68	8
mean±SD		42.1±9.6	1.83±0.85	1.01±0.54	0.61±0.32	0.14±0.09	0.05±0.03	0.03±0.03	2.37±0.57	28.6±14.9

WBC: white blood cell; Neu: neutrophil; Lym: lymphocyte; Mon: monocyte; Eos: eosinophil; Bas: basophile; RBC: red blood cell

**Table 3:** Baseline characteristics of healthy volunteers.

Patient no.	Gender	Age (years)	Total WBC count(×10^9^/L)	Neu	Lym	Mon	Eos	Bas	RBC count (×10^12^/L	Platelet count (×10^9^/L)
1	Male	25	6.82	4.65	1.73	0.34	0.06	0.04	4.68	139
2	Male	44	7.88	4.82	2.14	0.62	0.20	0.08	5.04	235
3	Female	38	5.83	3.52	1.61	0.52	0.12	0.06	4.26	156
4	Female	47	8.62	5.40	2.42	0.56	0.22	0.02	4.38	337
5	Female	33	7.19	4.39	1.70	0.63	0.30	0.17	3.97	226
6	Female	50	8.32	5.25	2.13	0.60	0.22	0.12	4.87	158
7	Male	52	9.09	6.53	2.06	0.34	0.16	0.00	5.23	219
8	Male	48	6.43	4.36	1.47	0.40	0.11	0.09	4.46	185
9	Male	33	8.11	5.43	2.12	0.34	0.18	0.04	4.24	207
10	Male	39	7.33	4.38	1.75	0.81	0.28	0.11	5.21	117
mean±SD		40.9±8.8	7.56±1.02	4.87±0.82	1.91±0.31	0.52±0.16	0.19±0.08	0.07±0.05	4.63±0.44	197.9±62.9

WBC: white blood cell; Neu: neutrophil; Lym: lymphocyte; Mon: monocyte; Eos: eosinophil; Bas: basophile; RBC: red blood cell

### Subject protection

This study, approved by the ethics committee of Shandong University, complied with the Declaration of Helsinki and Chinese law. All patients and healthy volunteers involved in this study provided written informed consents, signing their names in a document sent to the ethics committee of Shandong University.

### Diagnostic criteria

The Guide for Prevention and Treatment of SFTS published by the Ministry of Health of the People’s Republic of China in September 2010 was used to define the diagnostic criteria [1]. According to these guidelines, suspected cases involved epidemiological history (working, living, or travelling in forest zones, hills, or mountains during an epidemic season; documented tick bites 2 weeks before symptomatic onset), fever, gastrointestinal and neurological symptoms, and by laboratory tests revealing thrombocytopenia and leucopenia in peripheral blood. To confirm a case, at least 1 of the following conditions had to pertain: (1) positive nucleic acid identification of the newly identified bunyavirus in blood samples, (2) more than 4-fold increase in serum anti-bunyavirus immunoglobulin G titers during convalescence compared with acute phase, and (3) isolation of bunyavirus from the blood sample.

### Detection of bunyavirus nucleic acids

Methodologies described by Espy [2] and Mackay [3] were used for the detection of *bunyavirus nucleic acids*. All blood samples were transported immediately upon collection to the Virus Laboratory of the Center for Disease Control for laboratory tests.

### Bone marrow cytology

All 10 SFTS patients agreed for bone marrow collection at the peak of illness (about 4-5 days after disease onset), but only 4 patients allowed bone marrow biopsies, the other 6 or their families being reluctant, worried about bleeding of the biopsy site. Therefore, 4 bone marrow biopsies were carried out in healthy volunteers for controls. All bone marrow samples were collected aseptically at bedside with the posterior superior iliac spine chosen as biopsy site. Bone marrow smear (0.3 mL) were aspirated and spread uniformly on 6 slides, each 2×3 cm^2^, with just sufficient thickness to cover the slides. After samples were dried for 30 minutes at room temperature, Wright staining was performed, and stained slides microscopically evaluated by a trained technician. Bone marrow biopsies were collected at the same site, about 2-3 mm×10-12 mm marrow tissue was extracted and placed into a tube filled with 3-4 ml of Bouin solution for fixation. Then, tissues were dehydrated with high percentage ethanol and embedded into plastics (Hemapun 865 plastics). Sections were obtained with a microtome at a thickness of 3µm and slides submitted to Giemsa and Hematoxylin and eosin (H-E) stainings. Finally, sections were observed under light microscopy for hematopoiesis content, presence of fat and fibers, shape and number of marrow blood cells. 

## Results

### Baseline characteristics

The statistical analysis of baseline characteristics of the 10 SFTS patients in comparison with volunteers and acute aplastic anemia patients are shown in Tables 4 and 5, respectively. These data indicated that WBC and platelet counts in the SFTS patients were significantly reduced compared with that of volunteers, while no significant difference was observed in RBC count between these two groups. A comparison between SFTS and acute aplastic anemia patients showed no significant differences in WBC and platelet counts. However, RBC count was significantly higher in SFTS subjects than in acute aplastic anemia patients. 

**Table 4:** Statistical analysis of baseline characteristics of the 10 SFTS patients and 10 volunteers.

Parameter	t	p
WBC	13.28	0.00
Neu	10.54	0.00
Lym	9.13	0.00
Mon	9.12	0.00
Eos	4.86	0.00
Bas	3.35	0.01
Pla	7.78	0.00
RBC	1.52	>0.05(0.16)

WBC: white blood cell; Neu: neutrophil; Lym: lymphocyte; Mon: monocyte; Eos: eosinophil; Bas: basophile; RBC: red blood cell

**Table 5:** Statistical analysis of baseline characteristics of the 10 SFTS patients and 10 acute aplastic anemia patients.

Parameter	t	p
WBC	2.23	>0.05(0.06)
Neu	3.18	<0.05(0.01)
Lym	0.28	>0.05(0.79)
Mon	0.22	>0.05(0.83)
Eos	0.97	>0.05(0.36)
Bas	0.75	>0.05(0.47)
Pla	0.61	>0.05(0.55)
RBC	1.52	>0.05(0.16)

WBC: white blood cell; Neu: neutrophil; Lym: lymphocyte; Mon: monocyte; Eos: eosinophil; Bas: basophile; RBC: red blood cell

### Cytological examination of bone marrow smear

**Figure 1:**
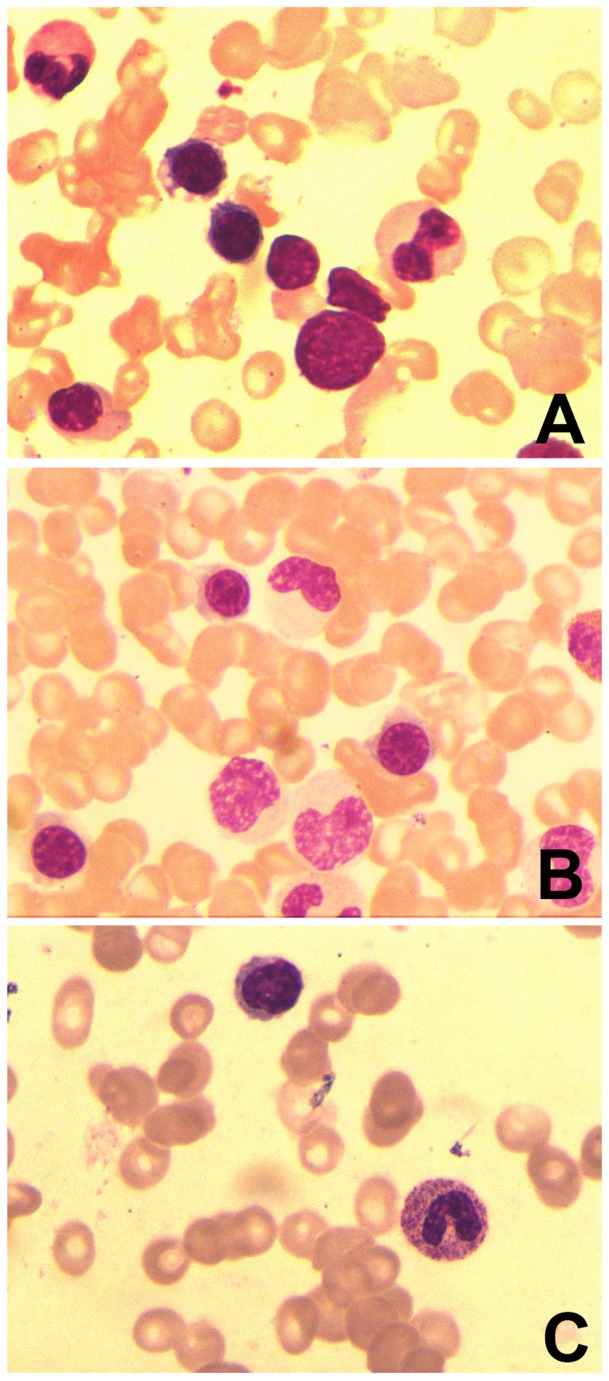
Bone marrow cytology image of aspiration smear. Bone marrow samples of aspiration from patients with SFTS (Figure 1 A), from healthy volunteers (Figure 1B) and from patients with aplastic anemia (Figure 1C).

Erythrocytes, megakaryocytes, and lymphocytes in bone marrow collected from the 10 SFTS patients displayed normal cell morphology, similar to healthy individuals. 12.3 ± 3.2 and 11.6 ± 4.2 megakaryocytes were counted per slide in samples from SFTS patients and healthy volunteers, respectively, a non- significant difference between these groups (t = 0.42, *P* >0.10). Only 1.6 ± 0.6 megakaryocytes were observed per slide in acute aplastic anemia patients, significantly lower compared with patients SFTS (t = 10.45, *P* < 0.0001). Representative micrographs of marrow cytology for the three groups are shown in Figure 1( Figure 1 A is the image of aspiration smear from patients with SFTS, Figure 1 B is that from healthy volunteers , Figure 1 C is that from patients with aplastic anemia) 

### Cytological examination of bone marrow biopsy samples

**Figure 2:**
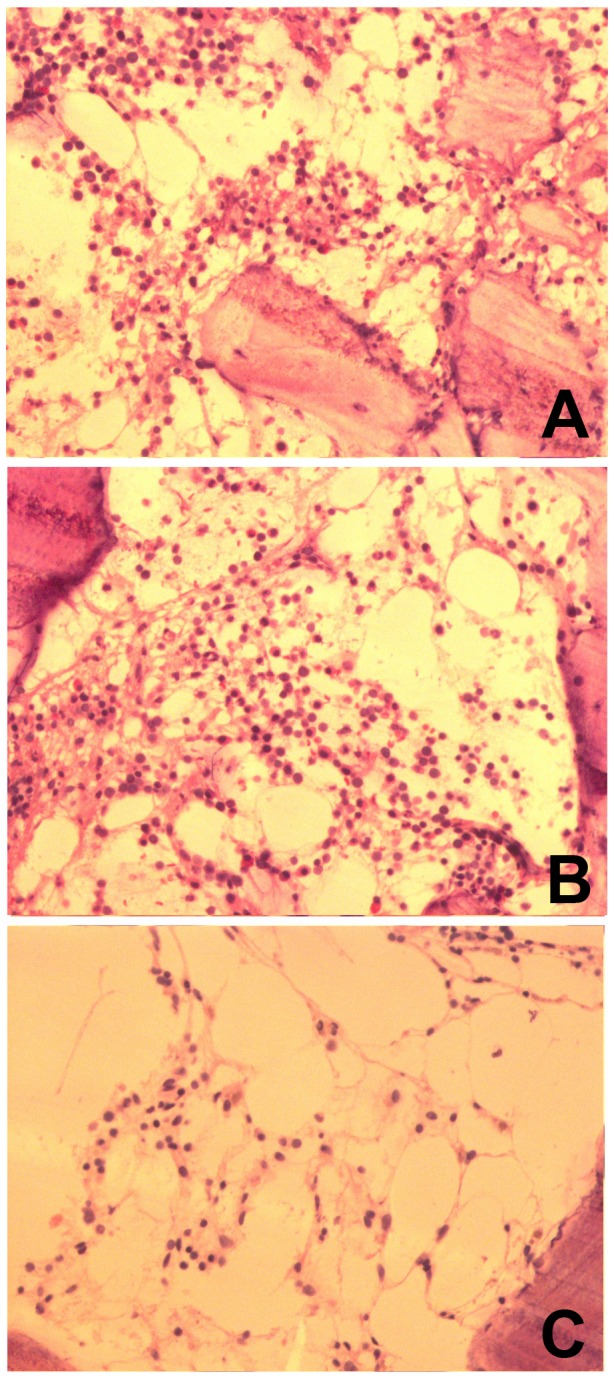
Bone marrow cytology image of biopsy. Bone marrow samples of biopsy from patients with SFTS (Figure 2A), from healthy volunteers (Figure 2B) and from patients with aplastic anemia (Figure 2 C).

The cytological characteristics of bone marrow from biopsy samples are summarized in Tables 6-8. Clearly, no significant differences were observed between the 4 SFTS patients and 4 volunteers: hematopoiesis, fat, and fiber contents, and megakaryocyte and plasmacyte numbers were similar(Table 9 are the statistical results). As shown in Figure 2, granulocyte, red blood and lymphocyte shapes were normal in SFTS patients(Figure 2 A)and volunteers(Figure 2 B).

**Table 6:** Cytological characteristics of bone marrow biopsy samples of the 4 SFTS patients.

Patient no.	Hematopoiesis content (%)	Fat content (%)	Fiber content (%)	Granulocyte (/mm^3^)	Red blood cell (/mm^3^)	Lymphocyte (/mm^3^)	Megakaryocyte (/mm^3^)	Plasmacyte (/mm^3^)
1	52.3	20.1	21.2	112.4	1435.6	65.8	16.4	18.5
5	46.6	23.8	25.1	98.4	982.4	90.6	12.6	25.3
6	60.1	18.1	18.6	160.2	675.9	89.8	15.3	20.2
9	43.6	27.3	25.6	178.4	1205.7	107.2	17.3	17.3
mean±SD	50.7±7,3	22.3±4.1	22.6±3.3	137.4±38.1	1074.9±324.0	88.4±17.0	15.4±2.0	20.3±3.5

**Table 7:** Cytological characteristics of bone marrow biopsy samples of the 4 volunteers.

Volunteer no.	Hematopoiesis content (%)	Fat content (%)	Fiber content (%)	Granulocyte (/mm^3^)	Red blood cell (/mm^3^)	Lymphocyte (/mm^3^)	Megakaryocyte (/mm^3^)	Plasmacyte (/mm^3^)
1	42.6	24.2	20.1	61.9	1613.6	48.6	13.4	17.2
2	45.6	21.7	20.3	168.6	896.4	94.3	16.5	20.4
3	50.2	20.2	21.5	143.5	783.9	93.8	12.7	18.3
4	46.8	27.3	18.1	136.4	1075.6	113.2	19.5	18.5
mean±SD	46.3±3.1	23.4±3.1	20.0±1.4	127.6±45.9	1092.4±367.7	87.5±27.4	15.5±3.1	18.6±1.3

**Table 8:** Cytological characteristics of bone marrow biopsy samples of the 10 acute aplastic anemia patients.

Patient no.	Hematopoiesis content (%)	Fat content (%)	Fiber content (%)	Granulocyte (/mm^3^)	Red blood cell (/mm^3^)	Lymphocyte (/mm^3^)	Megakaryocyte (/mm^3^)	Plasmacyte (/mm^3^)
1	18.6	55.1	31.9	40.3	453.2	67.4	2.5	18.6
2	20.2	47.3	27.2	27.3	209.4	60.6	3.3	20.6
3	15.7	50.4	27.6	30.2	354.1	87.0	3.3	25.1
4	23.5	39.0	29.7	56.0	245.3	90.8	4.2	19.0
5	18.4	40.3	35.6	22.9	190.6	54.7	1.5	16.8
6	27.3	41.5	22.5	18.6	170.3	108.2	1.3	21.3
7	21.3	45.2	26.5	37.9	398.5	110.3	2.3	22.5
8	16.1	38.4	32.0	10.6	403.7	68.3	1.8	19.5
9	17.4	43.2	34.5	54.2	502.2	77.9	1.6	17.2
10	23.5	39.5	30.0	30.1	324.7	82.5	1.4	16.4
mean±SD	20.2±3.7	44.0±5.5	29.8±4.0	32.8±14.6	325.2±116.4	80.8±18.8	2.3±1.0	19.7±2.7

**Table 9:** Statistical analysis of bone marrow biopsy’s cytological characteristics in SFTS and volunteers.

Parameter	t	P
Hematopoiesis content (%)	1.33	>0.05(0.27)
Fat content (%)	0.77	>0.05(0.50)
Fiber content (%)	1.16	>0.05(0.33)
Granulocyte	0.33	>0.05(0.74)
Red blood cell	0.07	>0.05(0.95)
Lymphocyte	0.06	>0.05(0.96)
Megakaryocyte	0.07	>0.05(0.95)
Plasmacyte	1.38	>0.05(0.26)

 Interestingly, significant cytological differences were found between bone marrow biopsies from the 4 SFTS patients and 10 acute aplastic anemia(Figure 2 C): hematopoiesis, fat and fiber contents, and granulocyte, red blood cell and megakaryocyte numbers were significantly altered. However, there were no significant differences in lymphocyte and plasmacyte numbers between the latter groups(Table 10 are the statistical results).

**Table 10:** Statistical analysis of bone marrow biopsy’s cytological characteristics in SFTS and acute aplastic anemia patients.

Parameter	t	P
Hematopoiesis content (%)	10.62	<0.05(0.00)
Fat content (%)	8.12	<0.05(0.00)
Fiber content (%)	3.17	<0.05(0.00)
Granulocyte	7.73	<0.05(0.00)
Red blood cell	6.64	<0.05(0.00)
Lymphocyte	0.70	>0.05(0.50)
Megakaryocyte	12.51	<0.05(0.00)
Plasmacyte	0.35	>0.05(0.73)

## Discussion

SFTS is a newly discovered disease with many unknown factors regarding pathogenesis and pathophysiology. Initially, SFTS was designated as human granulocytic anaplasmosis by clinicians. *Anaplasma* (a member of the *Rickettsia* family) was thought to be the etiological agent, and ticks the vectors. However, antibiotics effective against *Rickettsia* (such as tetracycline) were found not efficacious in the treatment of this disease. In 2011, after numerous studies, experts from the Institute of Virology of the Chinese Academy of Medical Sciences identified the pathogen as a novel virus, recently named bunyavirus, of which ticks appear to be the vectors [4]. SFTS is a systemic disease with multiple organ lesions and blood appears as the most damaged system. Since the use of antiviral drugs is ineffective in most cases, fatality rates are still considerably high [5,6]. 

 Clinically, it is believed that the main causes of peripheral blood thrombocytopenia and leucopenia include dysfunction of bone marrow regeneration, increased damage to the peripheral organs (such as spleen), and appearance of anti-platelet antibodies in the circulation. However, our data showed that bone marrow cytology was normal in SFTS patients, indicating an optimal bone marrow cell regenerative capacity. Thus, it is tempting to speculate that peripheral blood thrombocytopenia and leucopenia in SFTS patients result from increased peripheral organ damage or circulating anti-platelet antibodies. SFTS symptoms are markedly different from the manifestations of hemorrhagic fever with renal syndrome, caused by hantavirus (a bunyavirus subspecies), where thrombocytopenia in peripheral blood is a major phenotype, but with increased instead of reduced total white blood cell count [7]. The differences in etiology between SFTS and the disease caused by hantavirus requires further exploration. 
